# Effects of an educational intervention on oral hygiene and self-care among people with mental illness in Japan: a longitudinal study

**DOI:** 10.1186/s12903-017-0372-7

**Published:** 2017-04-27

**Authors:** Hatsumi Yoshii, Nobutaka Kitamura, Kouhei Akazawa, Hidemitsu Saito

**Affiliations:** 1 Department of Health Sciences, Tohoku University, Graduate School of Medicine, 2-1 Seiryo-machi, Aoba-ku, Sendai, Miyagi 980-8575 Japan; 2 Department of Medical Informatics, Niigata University Medical and Dental Hospital, Asahimachi-Dori 1-754, Niigata, 951-8520 Japan

**Keywords:** Oral health, Mental illness, Self-care, Education

## Abstract

**Background:**

The oral hygiene of patients with a mental illness is an important concern in psychiatric care, and it is necessary to increase the level of self-care among these patients. In this study, we administered an oral care questionnaire to people with mental illness in Japan and compared their answers before (baseline) and at 1 week and 1, 3 and 6 months after they participated in an educational program.

**Methods:**

The questionnaire was distributed to 390 patients. It included questions about age, education, income, between-meal snacks, number of teeth, frequency of tooth brushing, and other items. The educational program was developed for the purposes of improving self-care.

**Results:**

Before the program, the proportion of male patients who had had a mental illness for ≥ 10 years was significantly higher among those patients who did not brush their teeth before bed. In addition, such patients did not have primary care dentists, and a significantly higher proportion of male patients, compared with female patients, did not undergo routine dental checkups more than once per year. The educational program resulted in an improvement in the use of fluoride toothpaste from baseline to 6 months after the intervention (*p* = 0.001). The daily use of interdental brushes or floss was significantly different 6 months after the intervention.

**Conclusions:**

Male and long-term inpatients need oral hygiene instructions. Our educational program showed the effects of using oral hygiene tools. Future studies should include a control group to measure the impact of the educational program.

**Electronic supplementary material:**

The online version of this article (doi:10.1186/s12903-017-0372-7) contains supplementary material, which is available to authorized users.

## Background

The number of patients with mental illness in Japan is estimated to be 3,201,000, according to a survey conducted by the Ministry of Health, Labour and Welfare in 2011 [1]. This is equivalent to 2.5% of Japan’s total population of 127,799,000. In addition to taking antipsychotic drugs for their primary disease, many patients with mental illness take anti-Parkinson’s drugs for extrapyramidal symptoms because of the side effects of antipsychotic drugs. These patients may also take drugs for other complications. Consequently, patients often end up taking a combination of numerous drugs for their mental and physical health conditions. Because of drug-induced hyposalivation, the intraoral environments of these patients are characterized by an increase in the number of bacteria and caries [2]. In a review, Dougall et al. described the side effects of antipsychotic drugs and the related oral hygiene challenges among patients with mental illness [3]. Chu et al., in contrast, reported no association between second-generation antipsychotic drugs and the decayed, missing, and filled teeth (DMFT) index (beta = 0.03, *p* = 0.327) [4–7], suggesting that the incidence of side effects varies among drugs. Therefore, because patients with mental illness may need to take drugs for the rest of their lives, it is important to consider the side effects of these drugs that may cause serious problems in intraoral health.

In Spain, Arnaiz et al. reported that patients with schizophrenia had poor oral health compared with that of healthy individuals. Their study identified age and smoking status as factors influencing oral health (*p* < 0.001), and they reported correlations between negative symptoms and the number of lost teeth (*r* = 0.244, *p* < 0.05), DMFT index (*r* = 0.3527, *p* < 0.005), and community periodontal index (*r* = 0.533, *p* < 0.005) [8]. Surveys conducted in other countries have shown that oral hygiene status was worse in individuals with mental illness than in healthy individuals [4–7, 9].

Additionally, a survey on oral self-care conducted by Nielsen et al. in Denmark showed that patients with mental illness visited dental clinics less frequently than did healthy individuals [10]. The number of dental visits was particularly low among patients with intractable schizophrenia who were ≥ 50 years old, those who visited a psychiatric clinic every month, and those who were taking the atypical antipsychotic drug clozapine. Similarly, in the United Kingdom, McCreadie et al. reported poor oral self-care practices among patients with mental illness [11]. However, in their study of the elderly living in the United States, Janardhanan et al. [12] found no significant difference in dental visits between healthy individuals and patients with mental illness. Likewise, previous comparative studies on oral self-care practices comparing individuals with mental illness and healthy individuals have generated inconsistent results. Because the management of mental illness is given priority over physical health—and especially intraoral health—in the field of mental health care, the actual state of oral self-care among individuals with mental illness living in society is unclear. Therefore, it is important to elucidate the actual situation specific to Japanese patients.

In this study, to establish an effective oral health support system for patients, we conducted a survey of individuals attending psychiatric day care centers in Japan to examine oral self-care practices and personal preferences centering on between-meal snacks. We also developed an oral hygiene educational program and investigated its efficacy.

## Methods

### Procedure

By mail or by telephone, we asked the directors of psychiatric hospitals in Japan to participate in this study. After the directors agreed to participate by returning the letter of agreement, we sent them the consent forms, which were then distributed to individuals attending psychiatric day care centers. Each participant completed a consent form and returned it to us.

We first conducted a survey of knowledge and self-care related to oral hygiene. Second, all respondents viewed an educational presentation on oral hygiene. Third, after 1 week, the respondents again answered questions on knowledge and self-care related to oral hygiene. Fourth, after 1 month, the respondents answered the same questions. Fifth, after 3 months, the respondents again answered the same questions. Finally, the respondents answered the questions again after 6 months.

### Measures

The questionnaire comprised a pre-questionnaire and post-questionnaires (Additional file 1). The pre-questionnaire was a survey of basic demographic characteristics and self-care related to oral hygiene. Basic demographic characteristics were assessed with 17 items, including name, sex, age, education, height and weight. For age, the category “60s” includes all respondents aged 60 or older. Twenty self-care items were included, based on questions used by the Japan Dental Association [13]. These questions asked, for example, whether respondents were currently concerned about certain dental or intraoral conditions and how many teeth they had. The post-questionnaire included 15 items that were on the pre-questionnaire, as well as six new questionnaire items, such as “did you understand the instruction booklet?” and “how many times did you read the instruction booklet at home?”

### Educational program

We developed the educational program used in this study. It consisted of five units: (1) cause of tooth loss, (2) dental caries, (3) dental cleaning, (4) periodontal disease, and (5) routine dental checkups. The type of information provided was, for example, “Tooth loss is caused by cavities and periodontal disease in 90% of cases, so you need to prevent them,” “Dental plaque is a cause of cavities and periodontal disease,” and “It is more effective to use an interdental brush or floss along with a toothbrush.”

The educational program consisted of a slide show of 37 slides with narration and required 30 min to complete. The researcher delivered the presentation using this program. In addition, we created a booklet containing printouts of the slide show and accompanying narration. Photos of patients’ mouths found in the educational material were used with the permission by oral health association of Niigata prefecture in Japan.

### Statistical analysis

All analyses were performed using the Statistical Package for the Social Sciences (SPSS), Version 23.0. Cross-tabulation and the χ^2^ test were used to compare oral health among patients with different demographic characteristics, physical conditions, and snacking habits. Cross-tabulation and the χ^2^ test were also used to compare patients with different demographic characteristics in terms of snacking, smoking, and self-care. Dunnett’s test was applied to assess changes in self-care items across time points (baseline, after 1 week, after 1 month, after 3 months, and after 6 months). Statistical significance was defined as p <0.05 (α-level = 5%).

### Ethics approval and consent to participate

This study was approved by the Medical Ethics Committee of Tohoku University Graduate School of Medicine and 19 of the participating psychiatric hospitals. Each participant completed a consent form and returned it to us.

## Results

### Participants’ characteristics

We contacted 25 psychiatric day care centers to request their participation in the study. Of these, we received letters of agreement from 19 of the directors. In total, 390 patients with mental illness completed consent forms. For the baseline questionnaire, there were 323 valid responses. For the questionnaires administered 1 week, 1 month, 3 months, and 6 months after the educational program, there were 218, 226, 191, and 183 valid responses, respectively. There were 142 valid responses including all four time points. Drop-outs were patients who stopped the use of the psychiatric day care centers. Demographic characteristics of the participants are shown in Table 1.

**Table 1:** Demographic characteristics (*n* = 323)

	Number	Percent
Age		
20s	16	5.0
30s	49	15.2
40s	72	22.3
50s	84	26.0
≥ 60	102	31.6
Mental illness		
Schizophrenia	208	64.4
Mood disorder	75	23.2
Other disease	21	6.5
Unknown	19	5.9
Duration of mental illness		
< 1 year	5	1.5
1–2 years	17	5.3
3–4 years	28	8.7
5–10 years	61	18.9
> 10 years	212	65.6
Comorbidities		
Cardiovascular disease	13	4.0
Stroke	2	0.6
Diabetes	35	10.9
None	273	84.5

### Association between intraoral conditions and demographic characteristics, physical conditions, and snacking: pre-questionnaire

An analysis of 323 valid pre-questionnaire responses was conducted. Participants were asked to select “yes” or “no” for several intraoral conditions: swollen and flabby gums; number of teeth ≤ 19 or ≥ 20; pain; bad breath; and concern about speech, appearance, and occlusion.

The results of the univariate analyses for clarifying these characteristics are shown in Table 2.

**Table 2:** Association between intraoral conditions and demographic characteristics, physical conditions, and snacking, *n* (%) (*n* = 323)

	Swollen and flabby gums			Number of teeth			Pain			Bad breath			Concerned about speech			Concerned about appearance			Concerned about occlusion		
	Yes	No	*p*	≤19	≥ 20	*p*	Yes	No	*p*	Yes	No	*p*	Yes	No	*p*	Yes	No	*p*	Yes	No	*p*
Total length of hospitalization			0.179			0.001*			0.291			0.076			0.107			0.56			0.113
< 1 year	26 (8.0)	113 (35.0)		34 (10.5)	105 (32.4)		28 (8.7)	111 (34.4)		47 (14.6)	92 (28.5)		24 (7.4)	115 (35.6)		43 (13.3)	96 (29.7)		38 (11.8)	101 (31.2)	
≥ 1 and < 5 years	21 (6.5)	76 (23.5)		37 (11.5)	60 (18.6)		27 (8.4)	70 (21.6)		46 (14.2)	51 (15.8)		28 (8.7)	69 (21.4)		36 (11.1)	61 (18.9)		39 (12.1)	58 (18.0)	
≥ 5 years	10 (3.1)	77 (23.9)		49 (15.2)	38 (11.8)		17 (5.3)	70 (21.6)		30 (9.3)	57 (17.6)		20 (6.2)	67 (20.7)		27 (8.4)	60 (18.6)		30 (9.3)	57 (17.6)	
Side effect of drugs			0.55			0.007*			0.011*			0.663			0.204			0.036*			0.042*
Yes	11 (3.4)	61 (18.9)		17 (5.3)	55 (17.0)		24 (7.4)	48 (14.9)		29 (9.0)	43 (13.3)		20 (6.2)	52 (16.1)		31 (9.6)	41 (12.7)		31 (9.6)	41 (12.7)	
No	46 (14.2)	205 (63.5)		103 (31.9)	148 (45.8)		48 (14.9)	203 (62.8)		94 (29.1)	157 (48.6)		52 (16.1)	199 (61.6)		75 (23.2)	176 (54.5)		76 (23.5)	175 (54.2)	
Daily consumption of sweets			0.012*			0.019*			0.054			0.451			0.658			0.074			0.352
Yes	24 (7.4)	68 (21.0)		25 (7.7)	67 (20.7)		27 (8.4)	65 (20.1)		38 (11.8)	54 (16.7)		22 (6.8)	70 (21.7)		37 (11.5)	55 (17.0)		33 (10.2)	56 (17.3)	
No	33 (10.2)	198 (61.4)		95 (29.4)	136 (42.2)		45 (13.9)	186 (57.6)		85 (26.3)	146 (45.2)		50 (15.5)	181 (56.0)		69 (21.4)	162 (50.1)		74 (22.9)	160 (49.6)	
Smoking			0.823			0.004*			0.134			0.504			0.227			0.829			0.243
Yes	18 (5.6)	80 (24.8)		48 (14.9)	50 (15.5)		27 (8.4)	71 (22.0)		40 (12.4)	58 (18.0)		26 (8.0)	72 (22.3)		33 (10.2)	73 (22.6)		37 (11.5)	61 (18.9)	
No	39 (12.1)	186 (57.5)		72 (22.3)	153 (47.3)		45 (13.9)	180 (55.7)		83 (25.7)	142 (43.9)		46 (14.2)	179 (55.5)		65 (20.1)	152 (47.1)		70 (21.7)	155 (47.9)	

Cross-tabulation, χ^2^ test, *p < 0.05

When the yes/no groups were compared based on the number of teeth, the significant factors (cross-tabulation, χ^2^ test; *p* < 0.05) were total length of hospitalization, side effects of drugs, daily consumption of sweets, and smoking.

### Association of demographic characteristics with snacking and smoking: pre-questionnaire

On the pre-questionnaire, respondents were asked to select “yes” or “no” to indicate whether they smoked and whether their daily consumption of snacks included the following: sweets, fruits, ice cream, cola, and coffee with sugar. The results of the univariate analyses for clarifying these characteristics are shown in Table 3.

**Table 3:** Association of demographic characteristics with snacking and smoking, *n* (%) (*n* = 323)

	Daily consumption of snacks (multiple answers)									Smoking		
	Fruit			Ice cream			Cola					
	Yes	No	*p*	Yes	No	*p*	Yes	No	*p*	Yes	No	*p*
Gender			0.049*			0.012*			0.005*			0.001*
Male	48 (14.9)	154 (47.6)		50 (15.5)	152 (47.0)		64 (19.8)	138 (42.7)		126 (39.0)	76 (23.5)	
Female	41 (12.7)	80 (24.8)		46 (14.2)	75 (23.3)		21 (6.5)	100 (31.0)		99 (30.7)	22 (6.8)	
Dry mouth			0.045*			0.467			0.053			0.006*
Yes	44 (13.6)	87 (26.9)		36 (11.1)	95 (29.4)		42 (13.0)	89 (27.6)		80 (24.8)	51 (15.8)	
No	45 (13.9)	147 (45.6)		60 (18.6)	132 (40.9)		43 (13.3)	149 (46.1)		145 (44.8)	47 (14.6)	
Diabetes			0.289			0.159			0.467			0.001*
Yes	7 (2.2)	28 (8.7)		14 (4.3)	21 (6.5)		11 (3.4)	24 (7.4)		16 (5.0)	19 (5.9)	
No	82 (25.4)	206 (63.7)		82 (25.4)	206 (63.8)		74 (22.9)	214 (66.3)		209 (64.6)	79 (24.5)	

Cross-tabulation, χ^2^ test, *p < 0.05

When the groups with and without daily consumption of fruits were compared, the significant factors (cross-tabulation, χ^2^ test; *p* < 0.05) were gender and dry mouth. When the groups with and without smoking were compared, the significant factors (cross-tabulation, χ^2^ test; *p* < 0.05) were gender, dry mouth, and diabetes.

### Demographic characteristics affecting oral hygiene self-care practices: pre-questionnaire

On the pre-questionnaire, respondents were asked about the following oral self-care practices: tooth brushing after meals, during outings, and before bed; use of fluoride toothpaste; use of interdental brushes or floss; primary care dentist; reasons for dental visits; being too busy to see a dentist sometimes; and having at least one dental checkup per year. Respondents were also asked if they had had dental calculus removed or a teeth cleaning in the last year, if they had received proper brushing instructions at a dental clinic, and if they had family members or other familiar persons who were interested in dental health.

The results of the univariate analyses for clarifying these characteristics are shown in Table 4.

**Table 4:** Association between self-care and demographic characteristics, *n* (%) (*n* = 323)

	Gender			Mental illness				Duration of mental illness			Diabetes		
	Male	Female	*p*	Schizophrenia	Mood disorder	Other	*p*	< 10 years	≥ 10 years	*p*	Yes	No	*p*
Tooth brushing after meals			0.033*				0.703			0.005*			0.243
Every day	65 (20.1)	53 (16.4)		70 (21.8)	31 (9.6)	16 (5.0)		54 (16.7)	64 (19.8)		10 (3.1)	108 (33.4)	
Sometimes	92 (28.6)	53 (16.4)		97 (29.9)	33 (10.2)	16 (5.0)		41 (12.7)	104 (32.2)		15 (4.6)	130 (40.3)	
No	45 (13.9)	15 (4.6)		41 (12.7)	11 (3.4)	8 (2.5)		16 (5.0)	44 (13.6)		10 (3.1)	50 (15.5)	
Tooth brushing before bed			0.001*				0.854			0.022*			0.029*
Every day	106 (32.7)	90 (27.9)		125 (38.8)	46 (14.2)	25 (7.7)		78 (24.1)	118 (36.5)		14 (4.3)	182 (56.4)	
Sometimes	37 (11.5)	14 (4.3)		35 (10.8)	12 (3.7)	4 (1.2)		16 (5.0)	35 (10.8)		8 (2.5)	43 (13.3)	
No	59 (18.3)	17 (5.3)		48 (14.9)	17 (5.3)	11 (3.4)		17 (5.3)	59 (18.3)		13 (4.0)	63 (19.5)	
Routine dental checkup at least once per year			0.001*				0.995			0.393			0.407
Yes	51 (15.8)	52 (16.1)		66 (20.4)	24 (7.4)	13 (4.0)		32 (9.9)	71 (22.0)		9 (2.8)	94 (29.1)	
No	151 (46.7)	69 (21.4)		142 (44.0)	51 (15.8)	27 (8.4)		79 (24.5)	141 (43.6)		26 (8.0)	194 (60.1)	

Cross-tabulation, χ^2^ test, *p < 0.05

When the groups with and without tooth brushing after meals were compared, the significant factors (cross-tabulation, χ^2^ test; *p* < 0.05) were gender and duration of mental illness. When the groups with and without tooth brushing before bed were compared, the significant factors (cross-tabulation, χ^2^ test; *p* < 0.05) were gender, duration of mental illness, and diabetes. When the groups with and without at least one dental checkup per year were compared, gender was the only significant factor (cross-tabulation, χ^2^ test; *p* < 0.05).

### Education-related changes in self-care practices: pre- and post-questionnaires

The 142 valid responses for the entire study period were used to investigate the effect of education (Table 5). Dunnett’s test was applied to assess changes in self-care items over the assessed time points (baseline, after 1 week, after 1 month, after 3 months, and after 6 months). Use of fluoride toothpaste was a significant factor from baseline to all time points after the educational program (*p* < 0.001). Daily use of interdental brushes or floss was significantly different at 6 months after the educational program.

**Table 5:** Education-related changes in self-care practices (*n* = 142)

	Baseline	1 week	1 month	3 months	6 months	*p*			
Item	*n* (%)					Baseline vs. 1 week	Baseline vs. 1 month	Baseline vs. 3 months	Baseline vs. 6 months
Tooth brushing after meals									
Every day	55 (38.7)	52 (36.6)	51 (35.9)	54 (38.0)	52 (36.6)	0.933	0.838	0.999	0.933
Sometimes	62 (43.7)	64 (45.1)	60 (42.3)	61 (43.0)	64 (45.1)	0.992	0.992	0.999	0.992
No	25 (17.6)	26 (18.3)	31 (21.8)	27 (19.0)	26 (18.3)	0.998	0.408	0.970	0.998
Tooth brushing on the go									
Always	29 (20.4)	29 (20.4)	29 (20.4)	23 (16.0)	26 (18.3)	1.000	1.000	0.432	0.893
Sometimes	37 (26.1)	26 (18.3)	30 (21.1)	37 (26.0)	36 (25.4)	0.102	0.449	1.000	0.999
No	76 (53.5)	87 (61.3)	83 (58.5)	83 (58.0)	80 (56.3)	0.070	0.384	0.523	0.815
Use of fluoride toothpaste									
Yes	65 (45.8)	108 (76.1)	107 (75.3)	111 (78.1)	98 (69.0)	0.001*	0.001*	0.001*	0.001*
No	45 (31.7)	23 (16.2)	19 (13.4)	16 (11.3)	17 (12.0)	0.001*	0.001*	0.001*	0.001*
Unaware of toothpaste content	32 (22.5)	11 (7.7)	16 (11.3)	15 (10.6)	27 (19.0)	0.001*	0.003*	0.001*	0.675
Use of interdental brushes or floss									
Every day	21 (14.8)	29 (20.4)	27 (19.0)	28 (19.7)	32 (22.5)	0.154	0.382	0.250	0.025*
Sometimes	29 (20.4)	27 (19.0)	29 (20.4)	26 (18.3)	25 (17.6)	0.983	1.000	0.929	0.828
No	92 (64.8)	86 (60.6)	86 (60.6)	88 (62.0)	85 (59.9)	0.540	0.540	0.825	0.402
Frequency of visits to the dentist									
Go on a regular basis; When there is nothing to worry about	27 (19.0)	29 (20.4)	26 (18.3)	29 (20.4)	32 (22.5)	0.929	0.994	0.929	0.348
Go early; When you have a concern	31 (21.8)	29 (20.4)	36 (25.4)	24 (16.9)	32 (22.5)	0.988	0.757	0.494	0.999
When there is a worrying symptom	70 (49.3)	66 (46.5)	71 (50.0)	75 (52.8)	65 (45.8)	0.901	0.999	0.809	0.809
When there are multiple symptoms	14 (9.9)	18 (12.7)	9 (6.3)	14 (9.9)	13 (9.2)	0.710	0.532	1.000	0.997

*P*, difference within groups, Dunnett’s test, *p < 0.05

## Discussion

### Self-care, duration of mental illness, and preferences

Because of the side effects of drugs, such as hyposalivation and dry mouth, soft drinks containing sugar can easily cause dental caries in patients taking antipsychotic drugs. Smoking, a pernicious habit of many patients with mental illness, is also a factor contributing to the development of periodontal disease [14]. When these risk factors are combined with inadequate oral hygiene, intraoral environments start to deteriorate, potentially causing significant damage in the oral cavity. In this study, the proportion of patients who were men and had had a mental illness for ≥ 10 years was significantly higher among patients who did not brush their teeth before bed, indicating that gender and the duration of disease are factors associated with the lack of self-care. This is consistent with the association between the duration of mental illness and unhealthy teeth reported by Tang et al. in China [15]. Our study also revealed that male patients tended to have unhealthy preferences, such as a lack of daily consumption of fruits, the daily consumption of ice cream or cola, and smoking. In addition, men did not have primary care dentists, and, compared with female patients, a significantly higher proportion of male patients did not undergo routine dental checkups at least once per year. Similarly, Persson et al. reported poor oral hygiene among male outpatients in Sweden [16], suggesting that it is important to establish intervention programs focusing on dental self-care, diet, and regular checkups for male patients with a mental illness. Our results and previous studies have indicated that interventions should target men and those who have had a mental illness lasting many years.

### Length of hospitalization and intraoral conditions

In this study, a significant association was observed between having ≤ 19 teeth and ≥ 5 years of hospitalization. This is consistent with the results of a previous study showing a correlation between the length of stay in a psychiatric ward and the deterioration of intraoral health [4, 8]. In the acute phase of psychiatric illness, physicians have no choice but to admit patients because of the appearance of positive symptoms such as hallucinations and delusions. To manage these symptoms, both physicians and patients focus on the treatment. This phase is also characterized by the inability of patients to maintain sufficient personal hygiene and grooming activities, such as face washing, bathing, tooth brushing, and dressing. These patients therefore require the occasional assistance of health care professionals. However, it is extremely difficult to obtain help with oral hygiene and maintaining good intraoral conditions, especially for those who do not brush their teeth regularly and for patients with psychiatric illness in the acute phase. In a study of psychiatric inpatients in China, Tang et al. reported that 75.3% of these patients had dental caries, 50.4% needed tooth extraction, and 78.8% needed treatment, showing poor intraoral conditions [15]. Furthermore, in Israel, Ramon et al. found that the number of dental caries was higher among psychiatric inpatients (6.7 ± 6.9) than among healthy individuals (1.36 ± 2.0) [9]. Although we did not objectively examine the intraoral conditions of our patients or compare these conditions with those of healthy individuals, the data indicate an association between the healthy life span of teeth and prolonged hospitalization.

The severity of mental health symptoms is not the only factor that prolongs hospitalization. For example, in Japan, approximately 70,000 psychiatric inpatients with stable conditions are regarded as social hospitalization cases, because they have no place to go after leaving the hospital. For that reason, these patients remain in the hospital for an average of 300 days, which is longer than the mean duration of hospitalization in other countries [17]. However, regardless of the reasons for hospitalization, it is essential to maintain the oral health of inpatients. This conclusion is supported by the work of Gurbuz et al., who reported a correlation between the community periodontal index and tooth brushing (beta = 0.59, *p* = 0.02) among inpatients with schizophrenia in Turkey [18]. After the acute phase of disease characterized by positive symptoms, patients experience negative symptoms such as abulia and autosynnoia, which results in a significant loss of interest in personal hygiene. This is also the case among patients with mood disorders and late-life depression [19]. Patients with mood disorders have, among other things, an increased risk of caries, xerostomia, taste abnormalities, and bruxism [20]. Because of such symptoms specific to mental illness, the maintenance of oral and dental health among psychiatric inpatients has been a challenge across all countries despite the well-known importance of oral hygiene practices.

### Oral hygiene instructions for individuals with mental illness

Our educational program showed the effects of using oral hygiene tools. In a review published in 2016, Khokhar et al. [21] found no evidence from trials that oral health advice results in clinically meaningful outcomes among people with serious mental illness. We think this is because there is doubt about the utility and benefit of oral hygiene education targeted to individuals with mental illness because of uncertainty about the capabilities of these patients and about oral hygiene education itself. As for doubts about the capabilities of patients with mental illness, this attitude should be overcome from the perspective of normalization, and health care researchers need to work diligently to improve the self-care abilities of these patients. For concerns about oral hygiene education in general, the audiovisual learning model in Dale’s Cone of Experience may be effective [22].

In this study, we developed educational materials on oral hygiene for individuals with mental illness and examined the efficacy of these materials using audiovisual aids. Taking into account that individuals with mental illness generally have shorter attention spans and reduced capacity for thinking, we replaced written explanations in the educational materials with cartoons and photographic images as much as possible to reduce the burden to patients and to make the booklet as impactful as possible. Although we used the content of a preexisting instruction booklet on oral hygiene, we also made attempts to attract participants by including problems familiar to individuals with mental illness, such as the relationship between antipsychotic drugs and the oral environment, as well as the relationship between personal preferences, such as soft drinks and smoking, and periodontal disease or dental caries. We hope that the findings of this study will be useful for improving oral hygiene treatment worldwide.

## Conclusions

This study showed an association between demographic characteristics and both intraoral conditions and oral hygiene self-care practices. Male and long-term inpatients need to be provided with oral hygiene instructions. Our educational program could be useful in helping patients to improve their oral self-care. In future studies, the inclusion of a control group will help to provide more information about the impact of the educational program.

## Additional file

Additional file 1: Pre-questionnaire and post-questionnaire. (XLSX 22 kb)

## Abbreviations

DMFT index: Decayed missing, and filled teeth index
